# Patient characteristics, validity of clinical diagnoses and Outcomes Associated with Suicidality in Inpatients with Symptoms of Depression (OASIS-D): design, procedures and outcomes

**DOI:** 10.1186/s12888-023-05230-9

**Published:** 2023-10-13

**Authors:** Viktor B. Nöhles, Felix Bermpohl, Peter Falkai, Christine Reif-Leonhard, Frank Jessen, Mazda Adli, Christian Otte, Andreas Meyer-Lindenberg, Michael Bauer, Kerstin Rubarth, Ion-George Anghelescu, Dan Rujescu, Christoph U. Correll

**Affiliations:** 1 Department of Child and Adolescent Psychiatry, Charité Universitätsmedizin – Berlin, corporate member of Freie Universität Berlin and Humboldt-Universität zu Berlin, Augustenburger Platz 1, 13353 Berlin, Germany; 2 Department of Psychiatry and Psychotherapy, Charité Universitätsmedizin – Berlin, corporate member of Freie Universität Berlin and Humboldt-Universität zu Berlin, Campus St. Hedwig Hospital, Berlin, Germany; 3 Clinic for Psychiatry and Psychotherapy, Ludwig-Maximilians-University Munich, Munich, Germany; 4 Department of Psychiatry, Psychosomatic Medicine and Psychotherapy, University Hospital Frankfurt, Frankfurt am Main, Germany; 5 Department of Psychiatry and Psychotherapy, University of Cologne, Cologne, Germany; 6 Department of Psychiatry and Psychotherapy, Charité Universitätsmedizin – Berlin, corporate member of Freie Universität Berlin and Humboldt-Universität zu Berlin, Campus Mitte, Berlin, Germany; 7 Center for Psychiatry, Psychotherapy and Psychosomatic Medicine, Fliedner Klinik Berlin, Berlin, Germany; 8 Department of Psychiatry and Psychotherapy, Charité Universitätsmedizin - Berlin, corporate member of Freie Universität Berlin and Humboldt-Universität zu Berlin, Campus Benjamin Franklin, Berlin, Germany; 9 Department of Psychiatry and Psychotherapy, Central Institute of Mental Health, Mannheim, Germany; 10 Department of Psychiatry and Psychotherapy, University Hospital Carl Gustav Carus, Dresden, Germany; 11 Institute of Medical Informatics, Charité Universitätsmedizin – Berlin, corporate member of Freie Universität Berlin and Humboldt-Universität zu Berlin, Berlin, Germany; 12 Institute of Biometry and Clinical Epidemiology, Charité Universitätsmedizin – Berlin, corporate member of Freie Universität Berlin and Humboldt-Universität zu Berlin, Berlin, Germany; 13 Clinic for Psychiatry, Psychosomatics and Psychotherapy, Mental Health Institute Berlin, Berlin, Germany; 14 Department of Psychiatry and Psychotherapy, Medical University of Vienna, Vienna, Austria; 15 Psychiatry Research, The Zucker Hillside Hospital, Northwell Health, Glen Oaks, NY USA; 16 Department of Psychiatry and Molecular Medicine, Zucker School of Medicine at Hofstra/Northwell, Hempstead, NY USA

**Keywords:** Depression, Suicidality, Observational study, Treatment, Correlates, Diagnostic agreement, Moderators, Mediators

## Abstract

**Background:**

Suicidality, ranging from passive suicidal thoughts to suicide attempt, is common in major depressive disorder (MDD). However, relatively little is known about patient, illness and treatment characteristics in those with co-occurring MDD and suicidality, including the timing of and factors associated with the offset, continuation or reemergence of suicidality. Here, we present the background, rationale, design and hypotheses of the Patient Characteristics, Validity of Clinical Diagnoses and Outcomes Associated with Suicidality in Inpatients with Symptoms of Depression (OASIS-D) study, an investigator-initiated, observational study, funded by Janssen-Cilag GmbH.

**Methods/Results:**

OASIS-D is an eight-site, six-month, cohort study of patients aged 18-75 hospitalized with MDD. Divided into three sub-studies and patient populations (PPs), OASIS-D will (i) systematically characterize approximately 4500 consecutively hospitalized patients with any form of unipolar depressive episode (PP1), (ii) evaluate the validity of the clinical diagnosis of moderate or severe unipolar depressive episode with the Mini-International Neuropsychiatric Interview (M.I.N.I.) and present suicidality (at least passive suicidal thoughts) present ≥ 48 h after admission with the Sheehan-Suicide Tracking Scale (S-STS), assessing also predictors of the diagnostic concordance/discordance of MDD in around 500 inpatients (PP2), and (iii) characterize and prospectively follow for 6 months 315 inpatients with a research-verified moderate or severe unipolar depressive episode and at least passive suicidal thoughts ≥ 48 h after admission, evaluating treatment and illness/response patterns at baseline, hospital discharge, 3 and 6 months. Exploratory objectives will describe the association between the number of days with suicidality and utilization of outpatient and inpatient care services, and structured assessments of factors influencing the risk of self-injurious behavior without suicidal intent, and of continuous, intermittent or remitted suicidality during the 6-month observation period.

**Conclusion:**

Despite their frequency and clinical relevance, relatively little is known about patient and treatment characteristics of individuals with MDD and suicidality, including factors moderating and mediating the outcome of both MDD and suicidality. Results of the OASIS-D study are hoped to improve the understanding of the frequency, correlates and 6-month naturalistic treatment and outcome trajectories of different levels of suicidality in hospitalized adults with MDD and suicidality.

**Trial registration:**

NCT04404309 [ClinicalTrials.gov]

## Background

Major depressive disorder (MDD) is among the most common mental disorders worldwide and has been increasing in recent decades [1]. The lifetime prevalence of MDD exceeds 10% [2, 3]. MDD criteria include depressed mood, diminished interest or pleasure, weight or appetite increase or decrease, insomnia or hypersomnia, psychomotor agitation or retardation, fatigue or loss of energy, feelings of worthlessness or inappropriate guilt, diminished ability to think or concentrate, or indecisiveness, and recurrent thoughts of death or recurrent suicidal ideation [4].

Patients with MDD are more frequently female, older [5, 6] and most often cared for in primary care settings [7, 8], where most antidepressants, mostly selective serotonin reuptake inhibitors (SSRIs) [9, 10] are prescribed, with escalation to secondary or inpatient care in cases of more severe acute or dangerous clinical scenarios. MDD requiring hospitalization may follow those demographics but has also been associated with treatment-resistant depression, occurring in approx. 20% of people with MDD [11], presence of passive or active suicidal ideation [12, 13], and lifetime history of a suicide attempt, which is present in approximately 20% of people with MDD [14].

Suicide is a significant public health problem. The World Health Organization (WHO) has reported more than 700,000 deaths per year (1/100) worldwide due to suicide in 2019. Among 15–29-year olds, suicide is the fourth leading cause of death, and 58% of suicides occurred before the age of 50 years [15]. Suicide attempts are generally preceded by various forms of suicidal ideation. Often, national surveys do not measure passive suicidal ideation, but only active suicidal ideation [16–18]. One possible reason for this is that suicidality has historically been thought to progress along a continuum, from passive suicidal ideation (i.e., thoughts about death or a desire for death in general), to active suicidal ideation (i.e., thoughts of killing oneself) to suicidal plans, to suicidal behavior [19, 20]. However, recent studies suggest that there is no such continuum and that the association between passive suicidality and suicidal behavior and between active suicidality and suicidal behavior is comparable [12, 13, 19]. Therefore, studies must include passive suicidal ideation.

MDD is one of the disorders most strongly associated with suicidality [21]. For example, in patients with MDD, at least passive suicidality is present in about 50% [22], lifetime prevalence of suicide attempts is 31% [23], and lifetime prevalence of completed suicide is up to 10% [24–26], more than 20 times higher than in the general population [27]. In fact, in a meta-analysis, next to previous suicidal behaviors, severe depressive symptoms was the only other factor that was significantly associated with suicidal ideation, suicide attempt as well as death by suicide [28]. This high co-occurrence of depressed mood with suicidality justifies the inclusion of suicidal thoughts and actions as a criterion for depressive disorder in the Classification of Mental and Behavioural Disorders (ICD-10) [29] and Diagnostic and Statistical Manual of Mental Disorders (DSM-5) [4].

Few studies have examined features that are correlated with depression with and without suicidality, including worthlessness, guilt, despair, depressive and manic symptoms, inner restlessness and agitation, sleep disturbances, previous suicidal behavior, hopelessness, rumination, social withdrawal, lack of activity, crying, self-injurious behavior, feelings of loss of control, experiences of derealization, and course of depression [30–34]. Furthermore, individual studies have identified relatively few factors that can influence the course of depression with suicidality, e.g. severity of depression, comorbid disorders, including anxiety, hopelessness, anger, misuse of alcohol and drugs, and personality disorders [34–37].

A meta-analysis showed that among general practitioners, the rate of correctly confirmed MDD diagnoses by psychiatric interview was 47.3% [38], with an increased likelihood of suboptimal medication management in the absence of MDD. However, the fact that general practitioners do not have specialist psychiatric training may explain the low rate. To what degree psychiatric care practitioners and trainees who often make the first diagnoses during the evaluation in the emergency room prior to hospitalization have a higher concordance rate between the clinical and research interview-derived MDD diagnosis requires further study. Although isolated reports have found associations between younger patient age [39], and comorbid personality disorders [40, 41] with discordance between a clinical and a gold-standard research diagnosis of MDD, more extensive research is needed to identify which patient demographic, illness or treatment factors are associated with a discordant MDD diagnosis.

Moreover, while clinical correlates of suicidal thoughts and suicidal behaviors have been researched extensively in MDD, the pattern of remission, recovery and recurrence of suicidal thinking and suicide attempts after an inpatient admission for MDD has received little attention. Based on the association between the severity of MDD and prior suicidality, other psychiatric comorbidities and indicators of disease severity, it is reasonable to assume that the following characteristics may also predict persistence, recurrence and severity of suicidality. Such factors include presence [28, 36, 42, 43] and duration [44] of depressive symptoms, anxiety symptoms [28, 36, 45], anger and hostility [43], comorbid borderline personality disorder [35, 46] and substance use disorder [28], suicidality at the index episode [28, 36, 42, 47], and impaired quality of life [48, 49].

For diagnosed MDD, guidelines recommend psychotherapeutic interventions for the treatment of MDD, with cognitive-behavioral therapy (CBT) having the strongest evidence of effectiveness. Moreover, antidepressants are recommended for moderate to severe MDD. With regard to suicide risk in MDD, suicidality should be continuously assessed during treatment [50–53]. In cases of acute suicidality, the management plan should be based on the person's ability to consent to a no-harm agreement and on individual risk and environmental factors [51, 52]. Thereby, crisis intervention, such as hospitalization [53] and/or suicide-focused psychotherapy should be offered, and an emergency plan be developed with the patient [52]. In addition, antidepressant treatment is recommended for depressed patients with suicidality [51–53]. However, antidepressants are not recommended for suicidal crises because it takes up to 4 weeks for the antidepressant to take full effect [52, 54]. Benzodiazepines are recommended for short-term treatment to reduce, among other symptom domains, insomnia and agitation, which is also associated with a positive effect on acute suicidality [52, 55, 56], although their cost–benefit ratio is debated [57–60]. In psychiatric emergencies, especially in cases of acute suicidality, esketamine can be offered intranasally or ketamine intravenously in addition to an antidepressant [52, 61]. Moreover, intranasal esketamine has also shown efficacy for treatment-resistant depression, and is recommended by guidelines for this indication [52, 62–64]. Lithium can be offered when facing chronic suicidality [52, 65, 66]. As a last resort, electroconvulsive therapy can be offered in addition to psychotherapeutic crisis intervention in cases of acute suicidality [52, 55]. Understanding the factors that influence the trajectory of suicidal ideation and attempts in patients with MDD is crucial for enhancing the timing, sequencing and selection of targeted treatment strategies. Factors influencing the management plan include the severity of depressive symptoms [28, 36, 42, 43], comorbid disorders such as anxiety [28, 36, 43, 45] and personality disorders [35, 46], personality traits such as anger [43], and degree of quality of life impairment [48, 49]. Furthermore, the degree to which previous suicidal ideation and attempts can predict future suicidal ideation and attempts has been discussed [67–69]. However, a recent study did not support this connection, indicating that further research is required for verification or for a more precise delineation of patient subgroups who are at particularly high risk for recurrence or chronicity of suicidal thought and/or behaviors [70].

However, there remains a large information gap to better predict disease trajectories in patients with MDD and suicidality. Moreover, no standard of care treatments have been established and there is a need to explore new treatment options. The naturalistic Patient Characteristics, Validity of Clinical Diagnoses and Outcomes Associated with Suicidality in Inpatients with Symptoms of Depression (OASIS-D) study aims to improve the understanding of the frequency, correlates and 6-month naturalistic treatment and outcome trajectories of different levels of suicidality in hospitalized adults with MDD and suicidality. Such additional information can be used to guide clinicians toward focusing on specific patient subgroups at risk for more severe, chronic or dangerous suicidality and, possibly, devise a clinical standard of care for future comparator studies of novel agents aimed at reducing suicidality and its serious consequences in patients with MDD.

### Trial design

**Fig. 1:**
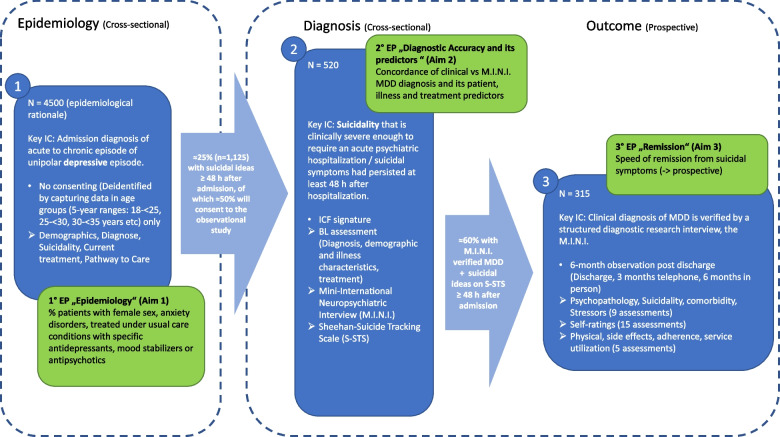
Study design of OASIS-D

OASIS-D has three different parts. Part one consists of a cross-sectional epidemiological chart review study in which basic historical and clinical characteristics, including past and current presence of suicidality, will be recorded in all consecutively hospitalized patients with MDD (patient population 1 (PP1)). Part two includes a consented subgroup of PP1 with suicidality that even ≥ 48 h post admission still presents with suicidality (eliminating transient, brief suicidality in response to an acute internal or psychosocial stressor) and that consents to undergo in-depth assessments of their past and present psychiatric history, psychiatric diagnoses and psychopathology, including suicidality (PP2). Part 3 includes a subgroup of PP2 whose MDD diagnosis and ongoing suicidality have been confirmed with research interviews and who are followed naturalistically for 6-months, reflecting usual care of adults with MDD and suicidality. Data collection will occur at inpatient admission (T0, epidemiological PP 1), at baseline in patients with ongoing suicidality, i.e. at least 48 h after T0 (T1; PP2 as a subgroup of PP1), and in PP3 (subgroup of PP2) at T1 (more detailed baseline assessments), discharge (T2), 3 months after T1 (T3) and 6 months after T1 (T4) (see Fig. 1). Due to the variability of hospital discharge (T2), the T2 assessment may occur after T3 (3 months) or concurrently with T4 (6 months).

### Objectives

The primary objective 1 of PP1 is to systematically characterize patients consecutively admitted to a psychiatric inpatient unit with any current form of clinically diagnosed depressive episode that is part of MDD, and not of bipolar disorder.

The primary objective 2 of PP2 is to evaluate the validity of the clinical diagnosis of moderate or severe unipolar depressive in inpatients using a gold-standard research interview, and the predictors of the diagnostic concordance/discordance.

The primary objective 3 of PP3 is to conduct a 6-month, prospective, naturalistic follow-up study in a subgroup of PP2 that has verified moderate to severe MDD and ongoing suicidality using structured assessments of the duration and severity of suicidality, the frequency of recurrence of suicidality as well as any significant moderators and mediators.

## Methods

### Study setting

The investigator-initiated OASIS-D study is coordinated by Charité—Universitätsmedizin, Campus Virchow-Klinikum, Department of Child and Adolescent Psychiatry, Berlin, Germany, and funded by Janssen-Cilag GmbH.

Patients are being recruited from the following eight adult mental health centers: Three centers at the Charité – Universitätsmedizin Berlin, i.e. (i) St. Hedwig Hospital, Department of Psychiatry and Psychotherapy, (ii) Charité Campus Mitte, Department of Psychiatry and Psychotherapy, and (iii) Charité Campus Benjamin Franklin, Department of Psychiatry and Psychotherapy, (vi) Clinic for Psychiatry and Psychotherapy at the Ludwig-Maximilians-University Munich, (v) Department of Psychiatry, Psychosomatic Medicine and Psychotherapy at the University Hospital Frankfurt, (vi) Department of Psychiatry and Psychotherapy at the University of Cologne, (vii) Department of Psychiatry and Psychotherapy at the University Hospital Carl Gustav Carus in Dresden, as well as (viii) Central Institute of Mental Health, Department of Psychiatry and Psychotherapy in Mannheim.

### Primary inclusion criteria

Inclusion criteria for PP1 are (i) male and female patients; (ii) aged between 18 and 75 years; (iii) admitted to a psychiatric inpatient unit, and (iv) having an ICD-10-based chart diagnosis of single depressive episode (F32) or recurrent depressive episode (F33).

Inclusion criteria for PP2 (originating from PP1) are (i) male and female patients; (ii) aged between 18 and 75 years; (iii) admitted to a psychiatric inpatient unit; (iv) having an ICD-10-based *clinical* diagnosis of a single or recurrent depressive episode of *at least moderate severity*, including single depressive episode, moderate episode (F32.1), single depressive episode, severe episode without psychotic symptoms (F32.2), single depressive episode, severe episode with psychotic symptoms (F32.3), recurrent depressive episode, currently moderate episode (F33.1), recurrent depressive episode, currently severe episode without psychotic symptoms (F33.2), and recurrent depressive episode, currently severe episode with psychotic symptoms (F33.3); (v) *clinically* diagnosed suicidality that persists for ≥ 48 h after inpatient admission; and (vi) sufficient German language skills to understand the purpose and procedures of the study and cooperate with the study procedures; and (vii) providing written informed consent to participate in the study.

Inclusion criteria for PP3 (originating from PP2) are (i) male and female patients; (ii) aged between 18 and 75 years; (iii) admitted to a psychiatric inpatient unit; (iv) having an ICD-10-based *research* diagnosis according to the Mini-International Neuropsychiatric Interview (M.I.N.I.) [71] of a single or recurrent depressive episode of *at least moderate severity* (for details, see PP2 above); (v) *research interview-based* suicidality with a score ≥ 1 on item 2 (passive suicidality) OR item 3 (active suicidality) using the Sheehan–Suicidality Tracking Scale (S-STS) [21]; (vi) written informed consent to participate in the study.

### Exclusion criteria

Exclusion criteria for PP1 are (i) psychiatric inpatients younger than 18 years and older than 75 years; (ii) no clinical diagnosis of a unipolar depressive episode; (iii) depressive episode in the context of bipolar disorder.

Exclusion criteria for PP2 and PP3 are (i) psychiatric inpatients younger than 18 years and older than 75 years; (ii) depression secondary to a physical illness, such as hypothyroidism or vitamin B12 deficiency, or substance use; (iii) severe physical illness symptoms that preclude participation in the study; (vi) unable to understand the study procedures; (v) unable to give informed consent; (vi) unable to give consent due to (limited) legal capacity.

### Aims and hypotheses

The primary aim in PP1 is to systematically characterize patient, illness, and treatment characteristics of consecutively admitted patients with a clinical diagnosis of a single or recurrent unipolar depressive episode.

#### Hypothesis 1

We hypothesize that MDD at inpatient admission is characterized by (i) most frequent referral by a primary care physician, (ii) female patient predominance of about 60% [5, 6], (iii) older patients aged approx. 45 years [5, 6], (iv) admission due to psychiatric emergency in approx. 25% of the sample, (v) occurrence of treatment-resistant depression in approx. 20% of the sample [11], (vi) occurrence of at least passive suicidal ideation in at least 50% of the sample [6, 72], (vii) lifetime history of a suicide attempt in approx. 20% of the sample [14], and (viii) preponderance of medication treatment with selective serotonin reuptake inhibitors (SSRIs) [9, 10].

#### Hypothesis 2

The primary aim in PP2 is to test the validity of the clinical diagnosis of at least moderately severe MDD in patients with clinically documented suicidality using the M.I.N.I. and compare predictors (diagnosis, demographic and illness characteristics, treatment) between patient groups with concordant and discordant clinical and research diagnosis.

Hypothesis 2A: We hypothesize that in < 50% of patients the clinical diagnosis will not be confirmed by the structured research diagnosis [38].

Hypothesis 2B: We hypothesize that the patient group with unvalidated vs validated clinical diagnosis of at least moderately severe MDD by the research interview compared will be associated with (i) younger age (less historical information and more dynamic emergence of (co)morbidities) [39], (ii) female sex (possible diagnostic bias), (iii) lower number of previous depressive episodes (less historical validation), (iv) lower number of index admission in the context of a psychiatric emergency (less historical validation), (v) higher number of comorbid personality disorders (more diagnostic overlap) [40, 41], and (vi) lower suicidal severity (less severe psychiatric condition).

#### Hypothesis 3

The primary aim of PP3 is a 6-month structured recording of the duration and frequency persistence and recurrence of suicidality and its correlates in 315 patients diagnosed with at least moderately severe MDD by the M.I.N.I. and with confirmed presence of at least passive suicidal ideas by the S-STS ≥ 48 h after inpatient admission.

Hypothesis 3A: We hypothesize that the time to remission of suicidal symptoms after the baseline is a major determinant of shorter overall duration of passive or active suicidal ideation (PASI) during the entire 6-month observation period.

Hypothesis 3B: We hypothesize that the rate of remission of suicidal symptoms after baseline is a major determinant of lower recurrence of PASI during the 6-month observation period.

Hypothesis 3C and 3D: We hypothesize that a longer total duration of PASI (Hypothesis 3C) and a greater risk of recurrence of PASI after complete remission (Hypothesis 3D) during the 6-month observational phase is associated with the following factors at time T0 (i.e., “moderators”): greater severity of (i) depressive symptoms [28, 36, 42, 43], (ii) manic symptoms (below the threshold of (hypo)mania as part of MDD), (iii) anxiety symptoms [28, 36, 45], (iv) anger and hostility [43], (v) quality of life impairment [48, 49], (vi) global symptoms of illness and (vii) suicidality [28, 36, 42, 47], (viii) higher number of lifetime depressive episodes, (viii) index admission as part of a psychiatric emergency, (ix) longer duration of current depressive episode [44], (x) higher non-adherence to pharmacological and non-pharmacological treatments, (xi) comorbid borderline personality disorder [35, 46], and (xii) comorbid substance use diagnosis [28].

Exploratory study aim 1 of PP3 is to describe the association between PASI and suicide attempts (S-STS item 14) as well as with the utilization of outpatient and inpatient care services during the 6-month observation period.

Exploratory hypothesis 1A, 1B and 1C: We hypothesize that a higher number of days with PASI (exploratory hypothesis 1A), a higher number of days with active suicidal ideation (exploratory hypothesis 1B) and a higher number of suicide attempts (exploratory hypothesis 1C) during the 6-month observation period are significantly associated with a higher number of (i) outpatient appointments, (ii) emergency department visits, (iii) inpatient hospitalizations, (iv) hospitalizations as part of psychiatric emergencies, (v) psychiatric hospital bed days, and (vi) suicide-related hospital bed days.

Exploratory study aim 2 of PP3 is to assess risk factors for different levels of active suicidality, consisting of active suicidal ideation (S-STS item 3), active suicidality with preparatory action (S-STS 12), self-injurious behavior without suicidal intent (S-STS item 13), or suicide attempts (S-STS item 14).

Exploratory study hypothesis 2: Active suicidality as defined above is associated with (i) slower rate of remission of suicidal symptoms after baseline, greater baseline severity of (ii) depressive symptoms, (iii) manic symptoms (below the threshold of (hypo)mania as part of MDD), (iv) anxiety symptoms, (v) anger and hostility, (vi) quality of life impairment (vii) global illness symptoms, and (viii) suicidality, (ix) higher number of lifetime depressive episodes, (x) duration of current depressive episode, (xi) index admission as part of psychiatric emergencies, (xii) higher non-adherence to pharmacological and non-pharmacological treatments, (xiii) comorbid borderline personality disorder, and (xiv) comorbid substance use diagnosis.

### Assessments and timeline

In PP1, sociodemographic, family, illness, and treatment-related data are collected to characterize patients with MDD at the time of inpatient admission (T0), i.e., age (coded in 5 year intervals), sex, ICD-10 F-diagnosis, current and lifetime suicidality, psychiatric emergencies, current and previous treatments, medication resistance, and nonadherence.

In PP2, sociodemographic, illness and treatment data (i.e., sex, Body-Mass-Index (BMI), family psychiatric disorders, education/ work, pathways to admission, F-diagnosis of ICD-10, illness duration, number of episodes, current and lifetime suicidality, psychiatric emergency, current and previous treatment, medication treatment-resistance and non-adherence, substance use) are collected at ≥ 48 h after inpatient admission (T1). M.I.N.I. will be used as an interview to determine the research diagnosis of at least moderately severe MDD, and S-STS will be used to assess suicidality.

In PP3, assessments are performed at time points ≥ 48 h after admission (T1), inpatient discharge (T2), 3 months after T1 (T3, by telephone), and 6 months after T1 (T3). The following investigator interviews and rating scales will be performed: For suicidality the S-STS, for depression the Montgomery-Asberg Depression Rating Scale (MADRS) [73], for global improvement change the Clinical Global Impression-Change (CGI-C) [74], for global severity of illness the Clinical Global Impression-Severity (CGI-S) [75], for imminent suicide risk the Clinical Global Impression of Imminent Suicide Risk (CGI-I), and for resolution of suicide risk the Clinical Global Impression of resolution of Suicide Risk (CGI-SR-R), for mania the Young Mania Rating Scale (YMRS) [76], for personal and social performance the Personal and Social Performance Scale (PSP) [77], for service use the Service Use and Resource Form (SURF) [78], for borderline disorder the ICD-10 borderline criteria (Table 1). Additionally, information obtained from patients regarding psychotropic medications (reason of initiation, discontinuation, switching, effectiveness, safety), electroconvulsive therapy, substance use, and presence of specific psychiatric emergency as reason for hospitalization. Furthermore, the following self-rating questionnaires will be obtained from the patients: Quick Inventory of Depressive Symptomatology-Self Report (QIDS-S) [79], Beck Depression Inventory-II (BDI-II) [80], Beck Anxiety Inventory (BAI) [81], Quality of Life in Depression Scale (QLDS) [82], European Quality of Life Group, 5-Dimension, 5-Level (EQ-5-DL) [83], 36-item Short-Form Health Survey (SF-36) [84], Work Productivity and Activity Impairment (WPAI) [85], Patient Health Questionnaire 9-item (PHQ-9) [86], Beck Hopelessness Scale (BHS) [87], Munich Chronotype Questionnaire (MCTQ) [88], State -Trait Anger Expression Inventory (STAXI) [89], Aggression Questionnaire-Buss Perry (AF-BP) [90]) (Table 1).

**Table 1:** Assessments and timeline

	**T0** **(Inpatient admission)**	**T1** **(≥ 48 h after T0)**	**T2 (Inpatient discharge)**	**T3** **(Month 3 after the 1st examination day of T1 (by telephone unless still hospitalized), if ≥ 2-week interval to T2)**	**T4** **(Month 6 after the 1st examination day of T1, if ≥ 2-week interval from T2)**
**Patient population 1 (data from routine clinical practice + clustering of data into 5-year age intervals with % data on each)**					
Age (coded in 5-year intervals)	+				
Sex	+				
Referral context	+				
ICD-10 F-Diagnosis	+				
Suicidality (current, lifetime)	+				
Psychiatric emergency	+				
Current and previous treatment (medication, psychotherapy, inpatient, electroconvulsive therapy)	+				
Treatment-resistant depression	+				
Medication nonadherence	+				
**Patient population 2**					
*Diagnosis*					
Mini-International Neuropsychiatric Interview (M.I.N.I.)		+			
Sheehan Suicidality Tracking Scale (S-STS)		+			
*Demographic and disease characteristics*					
Age		+			
Sex		+			
Body Mass Index (BMI)		+			
Family psychiatric disorders		+			
Education, employment		+			
Referral context		+			
ICD-10 F-Diagnosis		+			
Depressive episode (number, duration)		+			
Suicidality (current, lifetime)		+			
Psychiatric emergency (admission, current)		+			
Current and previous treatment (medication, psychotherapy, inpatient, electroconvulsive therapy)		+			
Treatment-resistant depression		+			
Medication nonadherence		+			
Substance use		+			
**Patient population 3 (M.I.N.I. + S-STS validated)**					
*Psychopathology, suicidality, comorbidity, stressors*					
Montgomery-Asberg Depression Rating Scale (MADRS)		+	+	+	+
Sheehan Suicidality Tracking Scale (S-STS)			+	+	+
Clinical Global Impression—Change (CGI-C)		+	+	+	+
Clinical Global Impression—Severity (CGI-S)		+	+	+	+
Clinical Global Impression of Imminent Suicide Risk (CGI-SR-I)		+	+	+	+
Clinical Global Impression of resolution of Suicide Risk (CGI-SR-R)		+	+	+	+
Mini-International Neuropsychiatric Interview (M.I.N.I.)					+
ICD-10: Borderline criteria checklist		+			
Young Mania Rating Scale (YMRS)		+	+	+	+
Personal and Social Performance Scale (PSP)		+	+	+	+
Previous and current substance use		+	+	+	+
Psychiatric emergency (since last visit, current)			+	+	+
*Self-ratings*					
Quick Inventory of Depressive Symptomatology-Self Report [QIDS-S]^38^		+	+		+
Beck Depression Inventory—II (BDI-II)		+	+		+
Beck Anxiety Inventory (BAI)		+	+		+
Quality of Life in Depression Scale (QLDS)		+	+		+
European Quality of Life Group, 5-Dimension, 5-Level (EQ-5-DL)		+	+		+
36-item Short-Form Health Survey (SF-36)		+	+		+
Work Productivity and Activity Impairment (WPAI)		+	+		+
Patient Health Questionnaire 9-item (PHQ-9)		+	+		+
Beck Hopelessness Scale (BHS)		+	+		+
Munich Chronotype Questionnaire (MCTQ)		+	+		+
State -Trait Anger Expression Inventory (STAXI)		+	+		+
Aggression Questionnaire—Buss Perry (AF-BP)		+	+		+
*Utilization of health services*					
Service Use and Resource Form (SURF)		+		+	+
Documentation of clinical routine and treatment (both outpatient and inpatient) - Medication (including reason for initiation, discontinuation and switching, effectiveness and safety - Electroconvulsive therapy		+	+	+	+
*Physical examination and side effects*					
Body Mass Index (BMI)		+	+		+
Adverse side effect of medication		+	+	+	+
*Adherence*					
Questionnaire for recording adherence		+	+	+	+

### Sample size and statistical analyses

Regarding PP1, the projected number of approximately *n* = 3,000 of the epidemiologic sample of hospitalized patients with MDD in the included age range (18–75 years) is based directly on the number of these patients in the eight participating study centers that were sampled in preparation of the study in 2017. Since only descriptive results are analyzed for patient population 1, no formal power analysis and case number calculation are provided. Descriptive statistics will be used to characterize a systematically and consecutively included epidemiologic sample of patients with MDD. Two interim analyses are performed using the same described descriptive statistics in PP1 at the epidemiologic sample size time points of 500 and 2000, respectively. These interim analyses are performed to identify potential data gaps and opportunities for the participating centers to review and address data capture and recording procedures of routine clinical information relevant to the characterization of patients hospitalized with MDD. Due to a higher observed patient dropout (30–35%) in PP3 between discharge and 6-month follow-up than projected in the initial protocol (20%), the subject number in PP3 was increased (see below) and the study duration was extended, which also provides more time for recruitment of patients into PP1, i.e., until the date of the last assessment of the last patient in PP3, increasing the projected number of patients to approximately *n* = 4500.

The hypotheses pertaining to PP1 will be analyzed exploratorily by using appropriate descriptive statistics, as well as univariate tests (chi-square tests).

Regarding PP2, based on clinical experience and questionnaire information from the recruitment centers as part of the design preparations, an estimated 33% of 3331 patients (*n* = 1,099) with a clinical diagnosis of at least moderately severe MDD are assumed to be still suicidal ≥ 48 h after admission. Approximately 60% of this group are expected to consent to participate in the interview-based study (and the naturalistic, 6-month follow-up study, should they also fulfill criteria for PP3, determined during participation in PP2). The following analyses of congruence between clinical diagnosis and research diagnosis (gold standard) of at least moderately severe MDD will take place:

- (a) two groups will be formed (primary analysis), i.e., positive vs. negative validation of the clinical diagnosis with the research-based interview M.I.N.I.,
- (b) use of descriptive statistics to estimate proportions of patients meeting the gold standard of research diagnosis and their confidence intervals (see a, hypothesis 2A), as defined by diagnostic concordance with the clinical diagnosis at the time of hospitalization; and.
- (c) to perform multiple mixed logistic regression analysis with backward elimination to compare patients with positively validated and non-validated at least moderately severe MDD (see a, hypothesis 2B), with random factors to account for clustering of patients in centers.

Regarding PP3, the OASIS-D study was initiated with the assumption that approximately 50% of the 520 patients surveyed in PP2 would not meet ICD-10 criteria for the inclusion criteria of at least moderately severe MDD, so that260 patients would be enrolled in PP3. Assuming a 20% drop out rate, this would yield n = 208 with data at 6 months. During the interim analysis 2, in September 2022, we noted a higher drop-out rate of 34% in PP3 than the 20% expected attrition rate at the time of the study design. Due to this finding, the number of patients in PP3 was increased to n = 315, so that with an attrition rate of 34%, the number of patients with data at month 6 would remain at n = 208. Since there was a higher than initially projected transition rate from PP2 to PP3, so that the n of patients entering PP2 did not need to be increased.

Hypotheses 3A and 3C will be analyzed by means of methods for count data, such as Poisson or Negative Binomial models, depending on whether overdispersion is present in the data or not. The dependent variable will be overall duration of PASI during the 6-month observation period and the independent variable of main interest will be time to remission of suicidal symptoms after baseline.

Hypotheses 3B and 3D will be analyzed by using Cox proportional hazard models, where the dependent variable will be time-to-first-recurrence of PASI and the independent variable of main interest will be time to remission of suicidal symptoms after baseline.

Further independent variables for regression analysis will include patient, illness, and treatment variables, including severity of depressive symptoms (MADRS, Quality of Life in Depression Scale (QIDS-S) [79]), manic symptoms (YMRS), anxiety (BAI), anger/hostility (STAXI, AF-BP), global illness symptoms (CGI-S), quality of life (EQ-5-DL), and suicidality (S-STS); presence of psychiatric emergencies; number of lifetime depressive episodes; duration of current depressive episode; nonadherence to pharmacological and nonpharmacological treatments; and comorbid borderline personality and substance abuse. Additionally, a random factor accounting for clustering of patients in centers will be added to the models.

Assuming a minimum requirement of 10 patients per predictor variable in regression analyses [91–93] and approximately 20 independent patient, illness, and treatment variables that would be tested as potential correlates of time to remission of suicidality, we estimated that a sample size of at least 200 would be required.

Additionally, as a secondary analysis, we will divide patients into two groups using the median time up to the first S-STS value of zero, which are either below or above the median time to first complete remission of suicidality.

Furthermore, comparisons between the slower and faster remitting group of continuous variables will be performed using multiple comparison procedures (MCPs) with 3 measurement time points (T1, T2, and T4). Because only the primary outcome and very limited other parameters are collected by telephone at T3, data at T3 are not included in these analyses. Because the timing at T2 (hospital discharge) is variable, and this timing is not independent of the outcome measured, the duration between T1 and T2 is included as a covariate in the analyses. Cross-group comparisons of dichotomized or categorical variables are performed using chi-square statistics. For ease of comparison, continuous outcomes, such as depressive symptoms and medication use are calculated. All analyses are two-sided, with alpha = 0.05. Due to the exploratory nature of the study, no adjustment for multiplicity between different endpoints is conducted. Hence, p-values and confidence intervals need to be interpreted in a hypothesis-generating manner. In addition, multivariable regression analysis with backward selection will be performed to identify independent moderators or mediators of continuous and categorical outcomes, respectively. Moreover, missing continuous data will be treated using multiple imputation using chained equations and random effects for the clustering of patients in different centers.

Among the exploratory objectives, the patient, illness, and treatment factors that influence the risk of different levels of active suicidality are identified: i) active suicidality (S-STS item 3), ii) active suicidality with preparatory action (S-STS item 12), iii) self-injurious behavior without suicidal intent (S-STS item 13), and iv) suicide attempt (S-STS item 14) during the observation period of inpatient treatment and within 6 months of study inclusion (T1). Target parameters i)-iv) are associated with slower initial resolution of suicidality (lower than median time to S-STS score = 0); and with: Depressive symptom severity (MADRS, QIDS-S), manic symptoms (YMRS), anxiety (BAI), anger/hostility (STAXI, AF-BP), and global illness symptoms (CGI-S); higher number of lifetime depressive episodes; more frequent psychiatric emergencies; longer duration of current depressive episode; non-adherence to pharmacological and non-pharmacological treatments; comorbid borderline personality; and substance abuse diagnoses.

All analyses will be performed as intent-to-treat analyses, independent of non-adherence with clinically prescribed treatments or drop out from clinical care.

### Recruitment and informed consent

Data of PP1 will be obtained via chart review from clinical routine data of patients with MDD (without informed consent) consecutively admitted as inpatients at the eight participating sites. If pseudonymous transmission is prohibited due to the lack of patient consent and the respective state law, the data will be transmitted anonymously, i.e. pooled in an excel file to the study coordinating center.

PP1 serves as a screening for the recruitment of PP2, i.e. individuals in PP2 are recruited from PP1, and individuals in PP3 are recruited from PP2, based on the respective in- and exclusion criteria. Prior to enrollment in PP2, each patient is informed by study staff about the nature, aims, expected benefits and potential risks of the study verbally and in writing. Patients must be given sufficient time and opportunity to decide about study participation. The written informed consent form must be signed by the patient and study staff.

### Data management

Data collection takes place via electronic CRFs. For this purpose, the study software secuTrial® of the company interActive Systems GmbH (iAS) is used. The study data are collected online/offline and transferred directly to the database of the study server that is housed at the Charite University medicine in Berlin, Germany. The data transfer between the workstation computer (in each recruitement center) and the study server takes place via a secured connection (SSL encryption), so that the transferred study data cannot be manipulated. The data are stored in the database (Oracle). At the end of the study, the database will be closed after all entries have been entered. These patient data are only stored pseudonymously. The unique assignment to the patient is done via a paper printout, which is filed in the study folder of the respective recruitment site. The originals of all central study documents including documentation sheets will be stored in the recruitment site for at least 10 years after completion of the study. Medical records, paper report forms and original data should be retained for the longest possible period allowed by each participating center.

In some hospitals, the transmission of PP1 data to the study center takes place anonymously for data protection reasons. For this purpose, anonymized pooled data will be transmitted to the coordinating study center in an excel file.

### Dissemination plans

Publication of study results will occur regardless of how the nature of the results. Besides poster and/or oral presentations at scientific meetings, at least one main publication related to each of the 3 study populations according to the respective objectives and hypotheses will be prepared and submitted. Secondary publications will also be prepared and submitted.

## Discussion

This observational OASIS-D study is designed to investigate the characteristics of adults hospitalized with MDD with a specific focus on the presence, correlates and course of suicidality. The first cross-sectional goal is the epidemiologic characterization of consecutively admitted patients with MDD across 8 major hospital centers in Germany. The second cross-sectional goal is the comparison of the clinical and research diagnosis of at least moderately severe MDD and suicidality in inpatients determining factors associated with concordance and discordance of the diagnoses. The main goal of the prospective study is the assessment of the duration of suicidality and the duration and frequency of recurrence of suicidality and its influencing factors in inpatients with at least moderately severe MDD followed prospectively for 6 months during their in- and outpatient treatment phases. Furthermore, we will investigate the relationship between suicidality and its course with naturalistic treatments, health care service utilization and outcomes in patients hospitalized with at least moderately severe MDD.

Although MDD is one of the most common mental disorders with a large burden of the disease [94, 95], there relatively limited attention has been paid to the epidemiologic characterization of MDD with vs without different levels of suicidality [28, 96, 97]. Moreover, the diagnostic validity of clinical diagnoses in recent field trials for DSM-5 has been low [98]. Additionally, although measurement-based care has been gaining traction, especially in MDD [99–101], diagnostic accuracy is indispensable for the appropriate implementation of measurement-based care. However, studies of diagnostic concordance of clinical diagnoses with research interview-based diagnoses are scarce that could inform ways to improve diagnostic accuracy and, ultimate, inform investigations of the relationship between diagnostic concordance and guideline-conforming treatment as well as effects on outcomes. In these regards, the OASIS-D study can make several contributions by characterizing patients with and without different levels of suicidality in naturalistic treatment settings and by identifying the rate and correlates of diagnostic concordance vs discordance in patients hospitalized with MDD.

Furthermore, although suicidality is common, especially in MDD, and associated with serious adverse consequences, including but not limited to mortality [28, 97, 102–104], until the recent approval of esketamine [105–112], all treatments for depressive symptoms associated with acute suicidal behavior in patients with MDD have been off-label [50, 113, 114]. Moreover, standard of care for suicidality in patients with MDD, as a common comparator of agents seeking regulatory approval for suicidality, are unclear [50, 94]. Treatment recommendations include the aggressive management of the underlying depressive episode, psychotics features, and of comorbidities, including substance abuse, which can all worsen suicidality and MDD outcomes [50, 94]. In this regard, the OASIS-D study will provide information on the naturalistic treatment and outcomes of research diagnosis-confirmed at least moderately severe MDD that is associated with and complicated by different levels of research-confirmed suicidality, investigating the speed and course of changes in suicidality in these patients as well as related factors.

Several limitations of the OASIS-D study need to be considered. First, OASIS-D is an observational study, not an intervention study. Consequently, treatments are based on clinical decisions that may vary from clinician to clinician and clinic to clinic. This heterogeneity may bias the analysis of treatment outcomes and factors. On the other hand, this factor increases the generalizability of the findings. Second, six of the eight participating centers are University-based clinics, which might influence the patient population in PP1 and, possibly diagnostic accuracy in PP2. However, most of the 6-month follow-up duration in the prospective study of PP3 will be in community outpatient settings, increasing the generalizability of those findings. Third, the data in PP1 are limited to routine clinical data only, as the goal is a large sample and consecutive inclusion of inpatients, which required anonymous data acquisition as part of a chart review, not requiring informed consent. This approach may lead to gaps in the data that relied on clinical documentation, as more extensive data collection beyond routine clinical data, was not possible. Fourth, speed of offset and timing and duration of recurrence of different levels of suicidality are a key outcome. Although for example continuous ecologic momentary assessment of suicidality would have been able to ensure more fine-grained assessments of suicidality trajectories [115, 116], for feasibility purposes this study relies on intermittent interviews (baseline, discharge, 3 months and 6 months) to assess the period incidence and prevalence of suicidality. This approach could lead to recall bias that may affect the data, even though we are using the S-STS as a structured interview in order to improve the quality of the data. Finally, although treatments and service utilization are also a focus of this study, we will rely on patient report, for these outcomes too, which similarly could be subject to recall bias. Nevertheless, structured interviews are employed to mitigate this effect.

On the other hand, strengths of the OASIS-D study lie in the characterization of a consecutive epidemiological sample of hospitalized patients with MDD from eight major psychiatric centers across Germany in analysis, the in depth assessment of the diagnostic accuracy of at least moderate MDD and passive or active suicidality, including correlates, as well as prospective observation of patients with a verified diagnosis of MDD and suicidality over a 6-month period with regards to the course of their suicidality, related treatment and outcomes.

Results of the OASIS-D study are expected to inform clinical care with regards to a more detailed understanding of risk factors for different levels of suicidality in hospitalized patients with MDD, correlates of diagnostic accuracy or imprecision, as well as the course and correlates as well naturalistic treatment effects and outcomes of suicidality in patients with moderate-to-severe MDD starting from inpatient and followed through outpatient settings.

## Data Availability

Not applicable.
